# Neutralizing monoclonal antibody in patients with coronavirus disease 2019: an observational study

**DOI:** 10.1186/s12985-022-01944-6

**Published:** 2022-12-15

**Authors:** Xuejiao Liao, Dapeng Li, Jie Liu, Zhi Liu, Zhenghua Ma, Jingke Dong, Xiangyi Yang, Dan Shu, Jing Yuan, Lei Liu, Zheng Zhang

**Affiliations:** 1 Institute for Hepatology, National Clinical Research Center for Infectious Disease, Shenzhen Third People’s Hospital, The Second Affiliated Hospital, School of Medicine, Southern University of Science and Technology, Shenzhen, 518112 Guangdong Province China; 2 Department of Neurology, Tianjin Medical University General Hospital, Tianjin, 300052 China; 3 Department of the Third Pulmonary Disease, Shenzhen Third People’s Hospital, The Second Affiliated Hospital, School of Medicine, Southern University of Science and Technology, Shenzhen, 518112 Guangdong Province China; 4 Shenzhen Research Center for Communicable Disease Diagnosis and Treatment of Chinese Academy of Medical Science, Shenzhen, 518112 Guangdong Province China; 5 Guangdong Key Laboratory for Anti-Infection Drug Quality Evaluation, Shenzhen, 518112 Guangdong Province China

**Keywords:** COVID-19, SARS-CoV-2, Antibody therapy, Delta variant, Clinical manifestation

## Abstract

**Background:**

Clinical data on patients infected with the severe acute respiratory syndrome coronavirus 2 (SARS-CoV-2) delta variant are limited, especially on clinical status after the application of antibody therapy.

**Methods:**

We evaluated clinical status in patients with the SARS-CoV-2 delta variant after BRII-196 and BRII-198 treatment in an infectious disease hospital in China. We collected data on clinical symptoms, laboratory tests, radiological characteristics, viral load, anti-SARS-CoV-2 antibodies, treatment, and outcome.

**Results:**

In mid-June 2021, 36 patients with delta variant infection were identified in Shenzhen. The most common symptoms at illness onset were cough (30.6%), fever (22.2%), myalgia (16.7%), and fatigue (16.7%). A small number of patients in this study had underlying diseases, including diabetes (5.6%) and hypertension (8.3%). The application of BRII-196 and BRII-198 can rapidly increase anti-SARS-CoV-2 IgG. The median peak IgG levels in the antibody treatment group were 32 times higher than those in the control group (*P* < 0.001). The time from admission to peak IgG levels in the antibody treatment group (mean: 10.2 days) was significantly shorter than that in the control group (mean: 17.7 days). Chest CT score dropped rapidly after antibody therapy, with a mean duration of 5.74 days from admission to peak levels.

**Conclusion:**

The results of this study suggest that the application of BRII-196 and BRII-198 antibody therapy improved clinical status in patients with SARS-CoV-2 delta variant infection.

**Supplementary Information:**

The online version contains supplementary material available at 10.1186/s12985-022-01944-6.

## Background

The pandemic of coronavirus disease 2019 (COVID-19) has brought huge challenges to global public health [1]. It has been more than 27 months since the COVID-19 outbreak, and under the remaining severe situation, mutant strains have become the focus of attention [2]. Indeed, mutation of the virus has led to regional outbreaks, and variants have the characteristics of a long incubation period, strong infectivity, and immune escape, which has increased the burden of global prevention [3, 4].

A previous study reported that the alpha (B.1.1.7) variant spreads 40–80% faster than the original strain, and the delta variant (B.1.617.2) has been suggested to be more transmissible than the alpha variant [4]. The delta variant was first identified in India in October 2020, spread rapidly throughout a mostly unvaccinated population in this country and caused massive numbers of cases, hospitalizations, and deaths [5]. The WHO data show that the delta variant of concern (VOC) has spread in 148 countries since its discovery [6]. The delta variant has a mean R_0_ of 5.08, which is nearly twice as high as that of the original strain [7]. Studies in Scotland show that the delta variant was mainly found in younger and more affluent groups [8]. Moreover, compared with the alpha VOC, the risk of hospitalization for COVID-19 in those infected with the delta variant is approximately double. Rapidly ramping up vaccine coverage rates, enhancing public health and social measures, and clarifying the clinical symptoms and effective treatment measures of delta variant infection are even more urgent and important. Vaccination can effectively reduce the risk of severe acute respiratory syndrome coronavirus 2 (SARS-CoV-2) infection and hospitalization [9, 10]. Moreover, the monoclonal antibodies have been approved under emergency use authorization to effectively treat infected out-patients with high risk of COVID-19 disease progression as well as to subjects for the pre- or post-exposure prophylaxis (prevention) of COVID-19 [11]. BRII-196 (amubarvimab) and BRII-198 (romlusevimab), a cocktail of two monoclonal antibodies targeting distinct epitopes in SARS-CoV-2 spike protein, demonstrated a significant reduction in hospitalization and death rate in non-hospitalized patients at high risk of clinical progression in a phase 3 study, and received biologics license application approval from The National Medical Products Administration (NMPA) of China in December 2021 [12].

In mid-June 2021, patients with delta variant infection in Shenzhen, China, were isolated and treated at the early stage. This study aims to describe the clinical manifestations, laboratory examination, radiological characteristics, and outcomes of patients with delta variant infection after the treatment of BRII-196 and BRII-198 combination.

## Methods

### Patients

In the current study, data were collected from all consecutively hospitalized patients in June 2021 at Shenzhen Third People’s Hospital. Our hospital is a tertiary referral hospital in southern China and the only hospital authorized by the government in Shenzhen City to admit COVID-19 patients. This study was approved by the Ethics Committee of Shenzhen Third People’s Hospital. Written informed consent was obtained from all patients.

In June 2021, patients infected with the SARS-CoV-2 delta variant were found on foreign flights, which triggered a small-scale clustered outbreak in the community. The real-time reverse transcription polymerase chain reaction method was conducted to detect the presence of SARS-CoV-2. The diagnostic criteria were in accordance with National Centers for Disease Control and Prevention of China (China CDC) recommendations [13, 14]. Samples identified as positive were reconfirmed by the key laboratory of the Shenzhen CDC.

### Clinical information

Data on demographics, disease history, vaccination history, patient signs, and presenting symptoms were obtained by epidemiological investigation. The following clinical information for patients with delta variant was obtained from a review of the hospital computer medical system: laboratory tests, radiological characteristics, treatment, and outcomes. According to a compassionate use manner, 27 patients at high risk of clinical progression received treatment of BRII-196 (amubarvimab) and BRII-198 (romlusevimab) combination (Brii Biosciences). Exclusion criteria was subject < 18 years, or with any unstable condition, significant history of allergies, or known allergies to the components. 9 hospitalized patients with the delta variant and without antibody therapy were selected as controls during the same period.

Disease severity was classified into the following categories according to the four severity grades from the “Chinese Clinical Guidance for COVID-19 Pneumonia Diagnosis and Treatment (7th edition)” published by the China National Health Commission [14]: (1) mild illness, patients with mild symptoms and without radiological evidence. (2) moderate illness, patients with fever, respiratory tract symptoms, and radiological evidence of confirmed pneumonia. (3) severe illness, patients with one of the following: (a) respiratory distress (≥ 30 breaths/ min); (b) oxygen saturation ≤ 93% at rest; (c) arterial partial pressure of oxygen / fraction of inspired oxygen ≤ 300 mmHg. (4) critical illness, patients with one of the following: (a) respiratory failure requiring mechanical ventilation; (b) shock; (c) other organ failure that requires intensive care unit (ICU). All discharged patients met uniform discharge standards: no fever for three consecutive days, improved respiratory symptoms, obvious recovery of acute lung lesions, and two negative SARS-CoV-2 test results, 24 h apart.

### Laboratory examination

Laboratory tests included lymphocyte count, white blood cell count, platelet (PLT), interleukin-6 (IL-6), D-dimer, viral load, and serum antibody titer (IgG). The qRT–PCR measurement method of SARS-CoV-2 detection has been described in detail [15]. Nasopharyngeal specimens collected during the patient's hospitalization were sent to the laboratory as a virus transport case. Total nucleic acid was extracted from the samples, and qRT–PCR was applied to measure the Ct value. We assessed anti-SARS-CoV-2 IgG using a commercial assay (Autobio Diagnostics, China). A cutoff value of 1.0 was defined as positive. The experiment was performed according to manufacturer instructions.

### Chest CT scan

The uCT 760 scanner (United Imaging, Shanghai, China) was used to perform high-resolution chest CT scans in the supine position at the end of inspiration. The following features of chest CT scans were recorded: ground-glass opacity, crazy-paving pattern, reticulation, honeycombing, parenchymal bands, consolidation, air trapping, and bronchiectasis. The distribution of lung lesions was described as peripheral, random, or diffuse. Each lung was divided into three regions: upper, middle, and lower regions. The upper region refers to the region above the tracheal carina, the middle between the tracheal carina and the inferior pulmonary vein, and the lower below the inferior pulmonary vein. A semiquantitative score was used to assess the degree of lung involvement [16]. The severity of the lung involvement in each lobe was graded from 0 to 5: no involvement defined as 0; lung involvement < 5% defined as 1; 5–25% defined as 2; 26–49% defined as 3; 50–75% defined as 4; and > 75% defined as 5. The total CT severity score was obtained by summing all lobe scores. Chest CT was performed every 3 days after admission.

### Statistical analysis

Statistical analysis was performed with R version 4.1.0 software (R Core Team, Vienna, Austria). Frequency and percentages were used to describe categorical variables. The median and interquartile range (IQR) or mean and standard deviation were used to describe continuous variables. Group differences were determined by student’s t-test or Mann–Whitney U test for continuous variables, and Chi-square test or Fisher’s exact test for categorical variables. A 2-sided *P* value less than 0.05 was considered statistically significant.

## Result

### Study population

In mid-June 2021, a total of 36 patients with delta variant infection were identified in Shenzhen (Table 1). The epidemiological survey revealed that 29 cases were imported from abroad; the other 7 patients were airport staff or close contacts of infected airport staff.

**Table 1:** Baseline demographics and clinical characteristics of 36 patients

Variable	Overall, N = 36	Control group, N = 9	AT group, N = 27	*P* value*
Age, median (IQR), years	35 (30–48)	26 (20–32)	36 (32–50)	0.01
Sex, male, n (%)	24 (66.7)	5 (55.6)	19 (70.4)	0.44
BMI, median (IQR)	23.4 (20.5–25.5)	19.9 (18.7–21.6)	23.9 (22.9–25.8)	0.01
Smoking, n (%)	11 (30.6)	3 (33.3)	8 (29.6)	> 0.99
Coexisting conditions, n (%)				
Hypertension, n (%)	3 (8.3)	0 (0.0)	3 (11.1)	0.56
Diabetes, n (%)	2 (5.6)	0 (0.0)	2 (7.4)	> 0.99
Vaccine, n (%)				0.77
No	29 (80.6)	7 (77.8)	22 (81.5)	
First dose	2 (5.6)	0 (0.0)	2 (7.4)	
Second dose	5 (13.9)	2 (22.2)	3 (11.1)	
Disease severity, n (%)				0.07
Mild	9 (25.0)	5 (55.6)	4 (14.8)	
Moderate	24 (66.7)	4 (44.4)	20 (74.1)	
Severe	3 (8.3)	0 (0.0)	3 (11.1)	
Hospital stay, median (IQR), days	23.5 (21.7–25.5)	20 (17.0–25.0)	24 (23.0–25.5)	0.05
Symptoms				
Cough, n (%)	11 (30.6)	1 (11.1)	10 (37.0)	0.22
Fever, n (%)	8 (22.2)	3 (33.3)	5 (18.5)	0.38
Fatigue, n (%)	6 (16.7)	2 (22.2)	4 (14.8)	0.63
Myalgia, n (%)	6 (16.7)	2 (22.2)	4 (14.8)	0.63
Expectoration, n (%)	5 (13.9)	2 (22.2)	3 (11.1)	0.58
Chest tightness, n (%)	5 (13.9)	0 (0.0)	5 (18.5)	0.30
Diarrhea, n (%)	4 (11.1)	1 (11.1)	3 (11.1)	> 0.99
Headache, n (%)	4 (11.1)	0 (0.0)	4 (14.8)	0.55
Nasal congestion, n (%)	3 (8.3)	1 (11.1)	2 (7.4)	> 0.99
Hyposmia, n (%)	1 (2.8)	0 (0.0)	1 (3.7)	> 0.99

*AT* Antibody therapy

*P values were calculated with Fisher's exact test, Wilcoxon rank sum test or Wilcoxon rank sum exact test

This study included 24 males (66.7%) and 12 females (33.3%). The median age of the patients was 35 (IQR, 30–48) years, and 29 (80.6%) were not vaccinated. A small number of patients in this study had underlying conditions, including diabetes (5.6%) and hypertension (8.3%). The numbers of patients with mild, moderate and severe illness were 9 (25.0%), 24 (66.7%), and 3 (8.3%), respectively. The median hospital stay was 23.5 days. As of August 16, 2021, all patients had been discharged. The most common symptoms at illness onset were cough (30.6%), fever (22.2%), and myalgia (16.7%) or fatigue (16.7%). 27 of 36 patients received antibody therapy of BRII-196 and BRII-198, and 24 of them (88.9%) had mild or moderate illness. Patients in antibody therapy group were elder and had a higher BMI compared with those in control group.

### Antibody and viral load

**Fig. 1:**
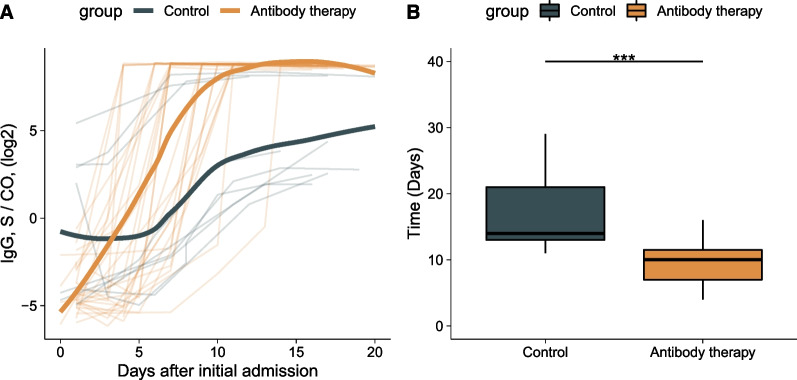
**A** Temporal profile of SARS-CoV-2 RBD-specific IgG antibody levels and **B** time from admission to peak value. The bold line indicates LOESS smoothing curve. ***, *P* < 0.001

**Fig. 2:**
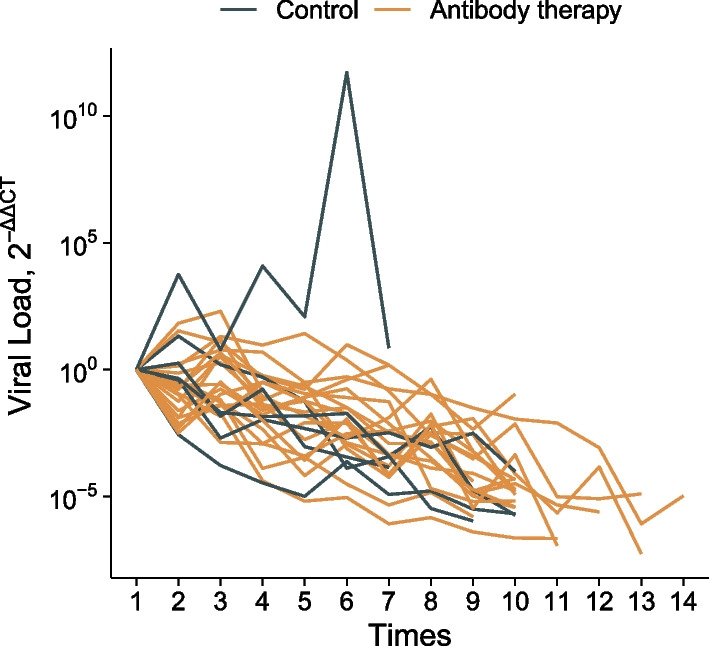
Change in SARS-CoV-2 viral load over time. Shown is the change in the individual's viral load relative to the baseline in 26 patients

In this study, 27 patients underwent antibody therapy. The median time of the use of BRII-196 and BRII-198 combination was at 5 days (IQR, 4–7 days) after admission, and 22 of the 27 patients (81.5%) were negative for anti-SARS-COV-2 IgG before treatment. Compared with those in the control group, IgG levels in the antibody therapy group rose rapidly up to a peak (Fig. 1A). The median peak IgG levels in the antibody treatment group [median IgG (IQR): 449.0 (440.5–458.9)] were 32 times higher than those in the control group [median IgG (IQR): 14.2 (7.3–271.9), *P* < 0.001]. The time from admission to peak IgG levels in the antibody treatment group (mean: 10.2 days) was significantly shorter than that in the control group (mean: 17.7 days; Fig. 1B). Viral load was measured in 26 patients after admission. Except for one patient who had a transient increase in viral load in the control group, all patients exhibited a gradual decrease in viral load after admission (Fig. 2). No significant difference in the viral load decline was found between the two groups.

### Changes in CT

Except for 3 patients with no lung involvement, most chest CT scans during the study showed bilateral lung involvement with peripheral distribution. Among them, 80.6% (29/36) of the patients had bilateral multiple lobe involvement; 75.0% (27/36) of the patient’s lung lesions were distributed around the periphery.88.9% (32/36) of the patients had ground-glass changes in the lungs. One patient with severe illness had diffuse multilobe involvement of ground glass opacities, reticulation, and parenchymal bands.

**Fig. 3:**
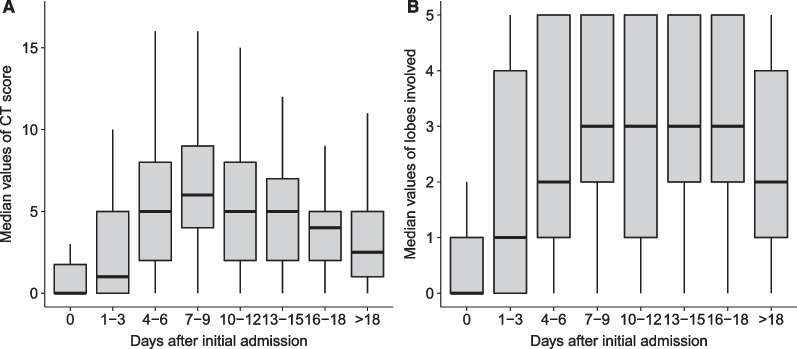
Temporal profile of **A** total CT scores and **B** the number of lobes involved. The box plot shows that median of the total CT score and the number of lobes involved

The total CT severity score at the time of admission ranged from 0 to 2 and reached a peak [median (IQR): 6 (4–10)] at 7–9 days after admission. At ten days after admission, the CT value showed a decreasing trend and decreased to 3 (IQR: 1–5) at the time of discharge. Moreover, the trend of the median number of lobe involvement was similar to that of the total CT severity score. The number of lobe involvement peaks [median (IQR): 3 (2–5)] was maintained at 7–9 days and decreased at 18 days after admission [median (IQR): 2 (1–4)] (Fig. 3). After treatment with antibody therapy, CT score dropped rapidly, with a mean duration of 5.74 days from admission to peak levels. There was no significant difference in the duration from admission to peak levels between the two groups.

### Biochemical changes

During hospitalization, the average of white blood cell, lymphocyte, and platelet counts were all within the normal range, with all showing an increasing trend. The average IL-6 level was in the normal range upon admission to the hospital (< 7 pg/ml), increased at 7–9 days (12.33 pg/ml), later exceeded the normal level, and gradually decreased to the normal range at 16–18 days (5.74 pg/ml) after admission. D-dimer levels on admission were normal (normal D-dimer level < 0.5 µg/mL). However, at 13–15 days after admission, the average D-dimer level had gradually increased and exceeded the normal range; when admission exceeded 18 days, the D-dimer level increased to 0.91 µg/mL (Additional file 1: Table S1).

## Discussions

This study reports clinical symptoms, biochemical tests, radiological characteristics, treatment and outcomes of 36 patients with laboratory-confirmed delta variant infection. The main symptoms of patients infected with the delta variant were cough, fever, myalgia and fatigue. In addition, patients without vaccination and a higher BMI were more likely to develop moderate or severe illness. Our results suggested that the BRII-196 and BRII-198 combination were safe and well tolerated, and delivered immediate IgG titers against the virus.

In 2021, the delta variant was the dominant variant of the disease worldwide. Compared with the original strain, the vaccine neutralization effect for the delta variant is significantly reduced [17]; another study reported that BNT162b2 can induce neutralization of B.1.617 and other SARS-CoV-2 variants [18]. Although the effectiveness of the vaccine is slightly reduced, it is currently believed that vaccination still effectively protects against infection by variants. The US Centers for Disease Control and Prevention (CDC) analyzed infection and hospitalization cases from May 1, 2021, to July 25, 2021, and revealed that unvaccinated individuals had a 4.9 and 29.2 times higher risk of infection and hospitalization, respectively [19]. Similarly, the proportion of moderate or severe disease in the unvaccinated group was increased in our study, which also indicates that vaccination remains the key to epidemic prevention.

In this study, BRII-196 and BRII-198 combination therapy was applied in a compassionate use manner. BRII-196 and BRII-198 are investigational neutralizing monoclonal antibodies that target distinct epitopes in the receptor binding domain of SARS-CoV-2 spike protein with extended half-life. The combination of BRII-196 and BRII-198 has been approved recently for treatment of COVID-19 in outpatients with high risk of COVID-19 disease progression by NMPA in China, and emergency use authorization (EUA) is being reviewed in US. The NMPA approval was based on final results from a Phase 3 study showing an 80% reduction in hospitalizations and deaths in the treatment group compared to placebo group [12]. A recent study, which enrolled patients with laboratory-confirmed SARS-CoV-2 infection and symptoms for up to 12 days, suggests potential heterogeneity in the treatment effect of BRII-196 plus BRII-198, possibly with a more favorable treatment effect in patients without endogenous neutralizing anti-SARS-CoV-2 antibodies [20]. In our study, a total of 27 patients treated with BRII-196 and BRII-198 at early stage exhibited improvement in pulmonary computed tomography imaging, with rapid elevations in IgG and without adverse events. The Omicron variant, which rapidly become dominant around the world, has raised concerns about the effectiveness of monoclonal antibodies approved for EUA [21]. An experimental study showed that the two subvariants of Omicron BA.1/BA.1.1 and BA.2 significantly reduced neutralization of most therapeutic antibodies. However, the BRII combo (BRII-196 + BRII-198), S309, and AZ combo (COV2-2196 + COV2-2130) maintained neutralizing activity despite the reduction [22]. A recent experimental study suggested that BA.4 and BA.5 were resistant to therapeutic monoclonal antibodies, except for Babtelovimab and Cilgavimab [23]. The effectiveness of therapeutic antibodies against the newest Omicron variants should be further investigated by clinical studies.

Although COVID-19 can cause multiple organ damage, SARS-CoV-2 mainly affects the respiratory system and can cause permanent damage to the lungs [24]. Previous research has shown that the lung lesions of patients infected with the original strain of SARS-CoV-2 were most severe at 13–15 days and gradually improved after a short plateau [16]. In the present study, CT results for patients infected with the delta variant were most severe at 7–9 days and then gradually improved. Moreover, CT score after antibody therapy dropped rapidly, with a mean duration of 5.74 days from admission to peak levels. Due to inconsistent discharge standards, the length of stay of patients with COVID-19 in different countries is not comparable. The median length of hospital stay in adults infected with the original strain in New York City was 14.77 ± 16.68 days [25]. A study from Beijing Ditan Hospital reported a length of hospital stay of 32.3 days and 21.7 days for imported and local cases, respectively [26]. Another study from Beijing found that the average hospital stay of patients infected with SARS-CoV-2 was 26.3 days [27]. In the present study, the median time of hospital stay of patients infected with the delta variant was 24 days after the treatment of BRII-196 and BRII-198 combination.

The clinical symptoms of COVID-19 vary, ranging from asymptomatic or mild to high inflammation states, which can cause acute respiratory distress syndrome and death in severe cases [28]. A previous study reported that SARS-CoV-2 infection leads to clinical deterioration by 7 to 10 days after initial presentation. A cytokine storm may occur, which is marked by elevations in IL-6, IL-10, TNF-a, and other inflammatory factors, as well as severe CD4+ and CD8+ T-cell lymphopenia and coagulopathy [29–31]. IL-6 affects cellular immunity with both proinflammatory and anti-inflammatory functions [32]. IL-6 was abnormally elevated at 7–9 days after infection in our study. Although IL-6 levels decline rapidly in the later stage, we should still be alert to the emergence of cytokine storms in patients infected with the delta variant.

There were some limitations in this study. First, although the results for clinical manifestations, viral load, and antibody tests represent encouraging findings, the study was limited by a non-randomized study design, a small sample size and an unbalanced control group. Therefore, it is challenging to determine the relationship between receiving antibody therapy and COVID-19 progression, and randomized control trials with large sample size are needed to verify these results. Second, all participants were enrolled from a single large city in China; therefore, the results of this study to other regions or regions with more severe cases, especially in those with limited resources, should be interpreted with caution.

## Conclusion

In summary, we report improving clinical status in patients with SARS-CoV-2 delta variant infection after the application of BRII-196 and BRII-198 combination. Most patients had mild or moderate symptoms, and all eventually recovered. Due to small sample sizes and non-randomized design, the effectiveness of monoclonal antibody treatment needs to be evaluated in large-scale clinical trials.

## Supplementary Information

**Additional file 1: Table S1.** Temporal profile of biochemical indicators.

## Data Availability

All datasets presented in this study are included in the article/supplementary material.

## Abbreviations

COVID-19: Coronavirus disease 2019
VOC: Variant of concern
SARS-CoV-2: Severe acute respiratory syndrome coronavirus 2
