# Supplementary material for: Neutralizing monoclonal antibody in patients with coronavirus disease 2019: an observational study
Source: Virol J. 2022 Dec 15;19:218. doi: 10.1186/s12985-022-01944-6 (PMC9753860; doi:10.1186/s12985-022-01944-6)
Supplement: Supplementary file 1 — Additional file 1: Table S1. Temporal profile of biochemical indicators. [file 12985_2022_1944_MOESM1_ESM.docx]

**Table S1** Temporal profile of biochemical indicators

| **Days after admission** | **LYMPH, 10^9^/L** | **WBC, 10^9^/L** | **PLT,**  **10^9^/L** | **IL-6,**  **pg/mL** | **D-dimer, ug/mL** |
| --- | --- | --- | --- | --- | --- |
| 0 | 1.09 (0.44) | 5.28 (1.51) | 189.54 (53.46) | 2.80 (9.16) | 0.41 (0.62) |
| 1-3 | 1.37 (0.87) | 5.47 (2.06) | 187.28 (61.92) | 4.65 (10.43) | 0.40 (0.47) |
| 4-6 | 1.50 (0.65) | 4.37 (1.53) | 163.91 (59.26) | 4.50 (9.84) | 0.57 (1.01) |
| 7-9 | 1.89 (0.83) | 5.24 (2.63) | 190.65 (76.58) | 12.33 (13.53) | 0.43 (0.31) |
| 10-12 | 1.82 (0.65) | 6.02 (2.18) | 237.27 (79.98) | 11.65 (10.28) | 0.43 (0.29) |
| 13-15 | 1.88 (0.59) | 6.18 (1.82) | 280.86 (76.48) | 9.57 (8.67) | 0.51 (0.44) |
| 16-18 | 2.07 (0.72) | 6.34 (1.52) | 273.41 (103.50) | 5.74 (4.91) | 0.82 (1.04) |
| >18 | 2.08 (0.90) | 6.30 (2.36) | 232.43 (74.32) | 6.06 (4.52) | 0.91 (2.13) |

Abbreviations: LYMPH, lymphocyte count; WBC, white blood cell count; PLT, platelet; IL-6, interleukin-6.
